# Palestine Is Freeing Us All Before Palestine Is Free: Comment on "The Rhetoric of Decolonizing Global Health Fails to Address the Reality of Settler Colonialism: Gaza as a Case in Point"

**DOI:** 10.34172/ijhpm.8677

**Published:** 2025-01-18

**Authors:** Yipeng Ge

**Affiliations:** Independent Researcher, Ottawa, ON, Canada.

**Keywords:** Liberatory Health, Indigenous Health, Settler Colonialism, Genocide, Palestine, Gaza

## Abstract

This commentary piece on the editorial piece by Eivind Engebretsen and Mona Baker entitled "The Rhetoric of Decolonizing Global Health Fails to Address the Reality of Settler Colonialism: Gaza as a Case in Point" explores the interconnected contexts of settler colonialism affecting health in occupied Turtle Island (also known as Canada) to Palestine. Addressing true and authentic health equity work means sharpening our politics and rhetoric beyond decolonizing "global health" as epistemically colonial and towards meaningful solidarity work and framings of liberatory or revolutionary health. There is a moral obligation to contextualize and historicize, rather than depoliticize. This ultimately means supporting through unwavering solidarity for collective liberation through the Indigenous resistance and resurgence movements in Turtle Island to Palestine if we are serious about decolonizing global health. Moving towards rhetoric and actions for collective liberation must be the focused goal of all who care deeply about true and authentic health equity work towards just and life-affirming systems for health for all.

 At a protest calling attention to the ongoing genocide in Gaza and ending Canada’s complicity in Israel’s genocide of the Palestinian people, there was a sign that read “Peace is the white man’s word, liberation is ours.” In this same vein, it is important to note that “global health” is the white man’s word, then what is ours? For those of us who seek to reimagine and co-create systems that do not dehumanize, nor subjugate people and entire communities, and do not wish to perpetuate the exact forms of oppression that health equity work demands of us, what is our word? To simply acknowledge the limitations of “global health” in examining inequities in health outcomes and how it is epistemically based in and rooted in colonialism is not sufficient. The calls for decolonizing “global health” will never be enough. We need new alternative rhetoric and tools to capture the necessary practices to dismantle and set us all free from the realities of genocide and colonialism. We need not to look far to understand that the answers lie in front of us. Indigenous Peoples (First Nations, Metis, Inuit) on occupied Turtle Island (also known as Canada) have been subjugated and dehumanized by an ongoing structure and process of colonization that has resulted in genocide and land theft. Respecting the self-determination of Indigenous Peoples and their sovereignty over their health and land is the only path forward.

 The authors of “The Rhetoric of Decolonizing Global Health Fails to Address the Reality of Settler Colonialism: Gaza as a Case in Point” propose an action-to-knowledge strategy as a method for dismantling the settler colonial infrastructure that is self-reinforcing in healthcare while grounded in the lessons and goals of grassroots social movements.^1^ However, what does this look like in practice moving forward for health equity work for which “global health” has evolved to capture and focus on in practice and application? Many including Dr. Ghassen Abu Sittah and Dr. Mads Gilbert have previously termed the alternative ideology as liberatory or revolutionary medicine. In the book, “*A Labour of Liberation*,” the author Dr. Baijayanta Mukhopadhyay writes that “healing work can be considered complicit with the task of reconciling yourself to the injustice of the world.”^2^ Before considering what liberatory or revolutionary medicine entails, we must contextualize how we as physicians or as “healers” reinforce the systems of oppression that we know contribute to disease and suffering by operating within these systems. The framing of “global health” plays into the existing landscape of structural injustice, allowing for the conditions for atrocities against humanity such as occupation, apartheid, and genocide to continue with impunity when health and wellbeing are depoliticized. The first step for authentic health equity work is to contextualize and historicize, rather than depoliticize.

 People and communities ultimately know the solutions to their complex challenges or at the very least the necessary conditions for their attainment of full and equal human rights. Communities understand the necessary changes in their conditions and determinants to heal from the colonial atrocities resulting in decades and generations-long material depravation and structural vulnerability. Human rights lawyer, Rabea Eghbariah, describes the Nakba as distinct from the legal concepts of apartheid and genocide in his piece, “The Ongoing Nakba: Towards a Legal Framework for Palestine.”^3^ Eghbariah writes that the “term Nakba, meaning ‘Catastrophe,’ is often used to refer to the making of the State of Israel in Palestine, a process that entailed the ethnic cleansing of over 750 000 Palestinians from their homes and destroying 531 Palestinians villages between 1947 to 1949. But the Nakba has never ceased; it is a structure not an event. Put shortly, the Nakba is ongoing.”^3^ Professor of international law, Dr. Ardi Imseis, also explained at the Committee on the Exercise of the Inalienable Rights of the Palestinian People at the United Nations that “the Nakba is a structure, not an event.”^4^ Colonization is structure and a process, not a single event in time. Decolonization is thus a reimagining of the future without the same tools that have maintained the structure and process of colonization.

 In this editorial piece, the authors Engebretsen and Baker highlight that there are limitations to a change in language alone with the “strong on rhetoric and weak on mounting a forthright challenge to the entire system supporting and perpetuating settler colonialism.”^1^ Recalling the foundational work and literature of Black radical feminist and activist, Audre Lorde, from her aptly named book and collection of essays entitled, *“The Master’s Tools Will Never Dismantle the Master’s House,”* the work of dismantling systems of oppression requires a new set of frameworks, tools, and rhetoric. The first step is to acknowledge through truth telling on the structural contributors that uphold a status quo that is deeply harmful to certain peoples and communities in this world steeped and dominated by white supremacy, structural racism, patriarchy, capitalism, and colonialism. Author and story-teller, Mathura Mahendren, has developed a toolkit, “Dismantling the Master’s Tools,” that feels more important than ever for how we consider the work ahead in decolonizing global health.^5^ A course facilitated by Abel Cano and Kortni Malone at the Harvard Kennedy School, “The Liberation Lab: A Course on Agency, Identity, and Restoration,” similarly applies the rhetoric of “the Master’s tools” to build a necessary restorative toolkit for what is ultimately decolonization work. One of the most important self-reinforcing tools of the colonizer or oppressor, is that of the colonized or oppressed mind. Our own embodiment of our intersectional identity and lived experiences of privilege and oppression often facilitate or limit how we develop our politics and show up for solidarity work. However, we must work to continually free ourselves from ways of thinking and doing that limit how we most authentically show up for health equity work through meaningful and sustained solidarity through action. In many ways, Palestine is freeing us all before Palestine is free.

 We must properly name and frame anti-Palestinian racism as a threat to health equity for Palestinians.^6^ Systemic racism and discrimination against Palestinians has shown us how Palestinian self-determination is an exception for global health institutions in confronting their own complicity in upholding structural violence by Zionist colonialism. Anti-Palestinian racism is a distinct form of racism that seeks to silence, exclude, erase, stereotype, and dehumanize Palestinians and their allies.^7^ The mischaracterization of voices in support of health and human rights for Palestinians often results in severe sanctions and disciplinary actions, a practice which has been advised against by the Office of United Nations High Commissioner for Human Rights.^8^

 The necessary context for understanding medicine as an institution that is deeply racist, violent, and extractive is important for decolonizing “global health.” There is a medical colonialism and apartheid legacy of eugenics and justification of slavery, forced sterilization, nutritional experimentation, forced displacement of Indigenous Peoples, and the list goes on. Communities and peoples, especially Indigenous Peoples, traditionally know the work of healing through deep care and love for one another and an understanding of the land and its gifts that help remedy and treat ailments of all sorts. The work and labour of healing have been co-opted by institutional professionalization and commodification under capitalism into what we understand now as “medicine.” In the book, *“Fighting for a Hand to Hold: Confronting Medical Colonialism against Indigenous Children in Canada,”* the author Dr. Samir Shaheen-Hussain lays out in stark and compelling detail the ongoing genocide of Indigenous Peoples in the settler colonial state of Canada and offers remedies for decolonizing healthcare. Dr. Shaheen-Hussain writes that “I don’t doubt the genuine intentions of most people who enter the health care field. Some of us are even fuelled by a burning desire for social justice. But we are not neutral. Claiming neutrality ignores history, politics, and the privileges that come – or don’t come – from one’s social position.”^9^ My own professional identity of being a trained doctor in Western biomedicine distances myself and others similarly trained in this way from our patients and communities reinforcing hierarchy and patriarchy which become inherent qualities of the system which we are taught as necessary evils. We are taught that the medical doctor holds the authority over the medical resources and knowledge, with the patient as the recipient. Medical doctors are taught to be knowledge keepers and gatekeepers of medical care and service providers of ‘medicine’ as a finite resource or good. In healthcare, we must move beyond these ways of thinking and doing. The acknowledgement for the right to health and the evolution of the social determinants of health to include structural determinants beyond access to healthcare services alone has allowed for continual shifting or our collective consciousness towards the true causes of the causes for disease and suffering. More rhetoric is focused on the commercial and colonial determinants of health in the hopes of more meaningfully addressing capitalism and colonialism as structures leading to poor health and death.^10^

 In the article, “A Collective Education Mentorship Model (CEMM): Responding to the TRC Calls to Action in Undergraduate Indigenous Health Teaching,” the co-authors including myself outline a model of decolonial education and teaching that was possible only through local community trust and enabled through the institutional framing of “global health” and “thinking global and acting local.”^11^ At this time, the Truth and Reconciliation Commission’s 94 Calls to Action were released as consequence to the testimonies of residential school survivors.^12^ The same year was the release of the “First Peoples, Second Class Treatment: The role of racism in the health and well-being of Indigenous Peoples in Canada” report by authors Dr. Billie Allan and Dr. Janet Smylie.^13^ The authors outline how “racism and colonization are inextricably intertwined” and that together they “deeply impact the health of Indigenous Peoples in Canada. Both in Canada and internationally, colonization has been recognized as a having a fundamental impact on the health of Indigenous Peoples.”^13^ The path forward for decolonizing efforts through liberatory or revolutionary health is unwavering support and solidarity for Indigenous Peoples’ inherent right to self-determination and sovereignty over their own health and healthcare provision from occupied Turtle Island to Palestine.

 The system of knowing and practice of “global health” is part of the colonial institutions that have been rooted in oppression, exploitation, and subjugation of Indigenous Peoples in the context of Turtle Island, and of Palestinians in the context of Palestine. Applying a decolonial lens means dismantling, and it means land back. It means reimagining and co-creating a present and a future beyond the existing colonial borders and frameworks. To practice liberatory or revolutionary health means to radically think about how our rhetoric and actions helps free more people from colonized and oppressed framings and mindsets and towards collective liberation through action. This means organizing for collective liberation through acts of meaningful solidarity, both big and small. For example, there have been over 3700 signatures from healthcare workers on so-called Canada who have signed onto a statement initially released November 10, 2023, titled “Urgent Statement Against the Israeli Destruction of the Health System in Gaza,” organized and led by Health Workers Alliance for Palestine that calls attention to the root causes of disease and suffering.^14^

 The efforts to incrementally change or reform “global health” so that it is more anti-racist or decolonial is important but in of itself will never be sufficient. We must move towards liberatory or revolutionary health. We must reckon with the contradictions in how we uphold structural oppression by operating within the existing systems while working to disrupt and dismantle them so we can reimagine and co-create life-affirming systems with and for communities who are made vulnerable by structural oppression leading and showing us the way. This means supporting in unwavering solidarity for collective liberation the Indigenous resistance and resurgence movements in Turtle Island to Palestine if we are serious about decolonizing “global health.” As activist, academic, and artist, Lilla Watson, aptly put, “If you have come here to help me, you are wasting your time. But if you have come because your liberation is bound up with mine, then let us work together.”^15^

## Ethical issues

 Not applicable.

## Conflicts of interest

 The author has worked as a volunteer doctor in Gaza, Palestine during the 2023-2025 genocide of the Palestinian people in Gaza by the Israeli forces. He, therefore, has an interest in ending the genocide as a first-hand witness.
